# Roles for IFT172 and Primary Cilia in Cell Migration, Cell Division, and Neocortex Development

**DOI:** 10.3389/fcell.2019.00287

**Published:** 2019-11-26

**Authors:** Michal Pruski, Ling Hu, Cuiping Yang, Yubing Wang, Jin-Bao Zhang, Lei Zhang, Ying Huang, Ann M. Rajnicek, David St Clair, Colin D. McCaig, Bing Lang, Yu-Qiang Ding

**Affiliations:** ^1^Department of Psychiatry, The Second Xiangya Hospital, Central South University, Changsha, China; ^2^National Clinical Research Center for Mental Disorders, Changsha, China; ^3^State Key Laboratory of Medical Neurobiology and MOE Frontiers Center for Brain Science, Institutes of Brain Science, Fudan University, Shanghai, China; ^4^Key Laboratory of Arrhythmias, Ministry of Education, East Hospital, Department of Anatomy and Neurobiology, Collaborative Innovation Centre for Brain Science, Tongji University School of Medicine, Shanghai, China; ^5^School of Medicine, Medical Sciences and Nutrition, Institute of Medical Sciences, University of Aberdeen, Aberdeen, United Kingdom; ^6^Department of Histology and Embryology, Institute of Neuroscience, Wenzhou Medical University, Wenzhou, China

**Keywords:** neocortex, IFT172, primary cilium, directed migration, corticogenesis

## Abstract

The cilium of a cell translates varied extracellular cues into intracellular signals that control embryonic development and organ function. The dynamic maintenance of ciliary structure and function requires balanced bidirectional cargo transport involving intraflagellar transport (IFT) complexes. IFT172 is a member of the IFT complex B, and IFT172 mutation is associated with pathologies including short rib thoracic dysplasia, retinitis pigmentosa and Bardet-Biedl syndrome, but how it underpins these conditions is not clear. We used the WIM cell line, derived from embryonic fibroblasts of *Wimple* mice (carrying homozygous Leu1564Pro mutation in Ift172), to probe roles of Ift172 and primary cilia in cell behavior. WIM cells had ablated cilia and deficiencies in directed migration (electrotaxis), cell proliferation and intracellular signaling. Additionally, WIM cells displayed altered cell cycle progression, with increased numbers of chromatids, highlighting dysfunctional centrosome status. Exposure to a physiological electric field promoted a higher percentage of primary cilia in wild-type cells. Interestingly, *in situ* hybridization revealed an extensive and dynamic expression profile of Ift172 in both developing and adult mouse cortex. *In vivo* manipulation of Ift172 expression in germinal regions of embryonic mouse brains perturbed neural progenitor proliferation and radial migration of post-mitotic neurons, revealing a regulatory role of Ift172 in cerebral morphogenesis. Our data suggest that Ift172 regulates a range of fundamental biological processes, highlighting the pivotal roles of the primary cilium in cell physiology and brain development.

## Introduction

The primary cilium, a microtubule-based structure that acts as the cell’s antenna and signaling hub (Berbari et al., 2009; Veland et al., 2014), originates from the centrosome (Paridaen et al., 2013) and is present on most growth-arrested or differentiated mammalian cells (Gerdes et al., 2009; Baudoin et al., 2012). Intraflagellar transport (IFT) is the evolutionarily conserved system that maintains the cilium’s structural and functional integrity. IFT protein complex A, together with dynein, is responsible for retrograde transport, while IFT complex B, with kinesin, facilitates anterograde cargo flow (Gerdes et al., 2009). Within the central nervous system, cilia are present on both neurons and glia, and their defective function results in various neurociliopathies (Valente et al., 2014) including Bardet-Biedel syndrome (BBS). Nevertheless, the precise role of ciliary function in brain development and homeostasis remains far from clear.

*Intraflagellar Transport 172 (Ift172)* is a gene encoding a member of IFT complex B (Follit et al., 2009). *Wimple* mice, carrying a homozygous *Ift172* point mutation (Leu1564Pro), were reported as embryonically lethal due to a number of developmental deficits (Huangfu et al., 2003). In humans, IFT172 mutation is associated with short-rib thoracic dysplasia (a skeletal ciliopathy) with or without polydactyly (Halbritter et al., 2013). Patients also present with retinal degeneration (Bujakowska et al., 2015) and Joubert syndrome-like cerebellar aplasia/hypoplasia (Halbritter et al., 2013). There have been case studies linking this gene to growth hormone deficiency (Lucas-Herald et al., 2015; Wit et al., 2016) and BBS (Schaefer et al., 2016), but how IFT172 is involved in these pathologies remains unclear.

Important ion channels, G-protein coupled receptors and key elements of many signaling pathways including Sonic Hedgehog and WNT, are concentrated in primary cilia (Nauli et al., 2003; Marley and von Zastrow, 2010; Murdoch and Copp, 2010; Sotak and Gleeson, 2012; Oh and Katsanis, 2013). Therefore, disruption of IFT integrity not only has a negative impact on the whole cilium (Gorivodsky et al., 2009) but also impairs signaling cascades essential for organogenesis and homeostasis (Huangfu et al., 2003; Haycraft et al., 2005; Huangfu and Anderson, 2005; Ocbina and Anderson, 2008; Zhang et al., 2012; Wang et al., 2013). In addition, members of the IFT family locate to the centrosome and Golgi apparatus (Jurczyk, 2004; Follit et al., 2009) and interact with the BBS complex – often referred to as the BBSome (Blacque et al., 2004; Wei et al., 2012; Sung and Leroux, 2013; Liew et al., 2014; Schaefer et al., 2016). The BBS complex also plays a role in IFT (Blacque et al., 2004) and, when defective, can result in BBS. IFT172 has been identified as the 20^th^ component of the BBS complex (Schaefer et al., 2016). Although it has been proposed to play a role in patterning the developing mouse brain and in the onset of BBS, there are no detailed mechanistic data available. Although WIM mutants and *Ift172*^–/–^ mice are available, they are embryonically lethal (Gorivodsky et al., 2009), and hence poor animal models for *in vivo* studies of the developing brain.

Therefore, we utilized mouse embryonic fibroblasts (MEFs) generated from *Wimple* mice (WIM) and their wild type (WT) counterparts to assess Ift172 control of cell migration and proliferation – key processes in brain morphogenesis. In addition, we manipulated the expression of Ift172 at critical stages of mouse brain development and determined the effects of *Ift172* silencing on corticogenesis using *in utero* gene transfer. We observed a spectrum of abnormal cell behaviors including perturbed cell proliferation and directed migration both *in vitro* and *in vivo*. We propose those deficits may lead to neuropathological changes that may underpin the development of neurociliopathies, including BBS.

## Materials and Methods

### Plasmids and *in utero* Electroporation

Two plasmids harboring mouse Ift172 shRNAs were purchased from Sigma (TRCN0000079813: 5′-CCGGGCGGCCATCAAC CACTATATTCTCGAGAATATAGTGGTTGATGGCCGCTTTT TG-3′; TRCN0000079814: 5′-CCGGGCTGCTGATCTCTCATT ACTACTCGAGTAGTAATGAGAGATCAGCAGCTTTTTG-3′). They contained the TRC2-pLKO-puro vector backbone with an insertion of the corresponding shRNA sequence (designed by the Broad Institute) and presented a knock-down efficiency of 94 and 91% as per the Sigma datasheet (Supplementary Table 1). They were abbreviated respectively as shRNA813 and 814 in the subsequent procedures. The control plasmid was also from Sigma and contained a non-specific sequence for any mammalian gene (SHC002; 5′-CCGGCAACAAGATGAAGAGCACCAACTCGA GTTGGTGCTCTTCATCTTGTTGTTTTT-3′).

Time-mated pregnant mice (E15.5) were anesthetized and embryos were manipulated surgically as described previously (Lang et al., 2016). Plasmids containing Ift172 shRNA 813 or 814 (0.5 μg/each) were injected into the lateral ventricles of the embryonic brains through glass micropipettes. CAG-EGFP was also injected (1 μg/μL) simultaneously to trace the cells transfected successfully. Five square electric pulses (30 V) were delivered through the uterus at 1 s intervals with forceps-type electrodes while the uterus was kept wet with saline. The female mice were then divided randomly into three groups. In group one, the mice received a single pulse of BrdU injection (100 mg/kg) on the next day after electroporation and the embryonic brains were harvested (E16.5) 1 h later. In groups two and three the pup brains were collected on postnatal day (P) 0 or 7 for histochemical examination. All the animal care and experimental protocols were reviewed and approved by the ethical committees of Tongji University Medicine School, Shanghai. All the experiments were performed in accordance with the relevant guidelines for the care and use of laboratory animals.

### Electrotaxis Assay

Direct Current Electric Field experiments were performed as described previously (Pruski et al., 2016), with ciliary abundance being assessed using the ciliary orientation protocol but without any serum starvation. Briefly, a chamber was made in a 96 mm tissue culture dish using no. 1 coverslips secured with adhesive silicone (Dow Corning RTV 3140), which was left to cure at least overnight before use. Small dams made from silicone compound (Dow Corning, DC4) restrained the pools of medium. 40 min before the start of the experiment cells were plated at a density of 20000 cells/cm^2^ into the central trough of the 1 cm × 4 cm chamber and a third coverslip was secured to the top using DC4 silicone compound. The cells were kept in 6–8 ml DMEM supplemented with 10% FBS and 2% 1M HEPES buffer. Time lapse migration experiments were done at 37°C using a microscope incubation chamber. The electric field was applied to the chamber via agar salt bridges (1% w/v agar in Steinberg’s solution: 58 mM NaCl, 0.67 mM KCl, 4.6 mM Trizma Base, 0.44 mM Ca(NO_3_)_2_, 1.3 MgSO_4_, pH 7.9). One end of each bridge was immersed in the medium in the chamber and the other end was in a beaker of Steinberg’s solution with a Ag/AgCl electrode connected to a DC power supply in series with a variable resistor. An electric field of 400 mV/mm was applied for 3 h.

Control cultures were treated identically, but were not connected to a power supply. Each hour the voltage was checked, adjusted if needed, and 1 ml of medium was exchanged for fresh medium from the anodal side. Images were collected at 10 min intervals with a 10x objective of a Nikon Eclipse TE2000-U microscope, SimplePCI software Version 5.3.1 (Compix, Inc.) and were analyzed using ImageJ software (National Institutes of Health, United States). The Manual Tracking (Institut Curie, France) and Chemotaxis (Ibidi GmbH, Germany) plug-ins were used to track and quantify cell migration. The x-Forward Migration Index is defined as the displacement distance along the x-axis (parallel to the electric field vector) divided by the total distance traveled.

### Immunocytochemistry

For immunocytochemical analysis of the cytoskeleton cells were plated at a density of 40000 cells/cm^2^ on glass coverslip (VWR) in a 4 well plate (Nunc, Nunclon). For cilia staining, cells were plated at 50000 cells/cm^2^ and after 22 h medium was changed to serum free medium. Cells were fixed using 4% paraformaldehyde on ice and subjected to primary antibody incubation overnight at 4°C. The primary antibodies were: anti-Arl13b (Proteintech, 1:1000), anti-acetylated α tubulin (Sigma, 1:10000), anti-α tubulin (Sigma, 1:10000), anti-pericentrin (Abcam, 1:1000) and anti-phosphorylated histone 3 (Cell Signalling, 1:1000). Secondary antibody and Hoechst (1:2000, Molecular Probe) incubations were done in 2% donkey serum in PBS at room temperature for 1.5 h. The secondary antibodies were: Alexa Fluor 488 (1:500, Molecular Probe), Alexa Fluor 594 (1:1000, Molecular Probe).

### Cell Culture and Immunoblotting

WIM cells and control WT cells obtained from C3H/HeJ background mice were kindly provided by Prof. Tamara Caspary (Emory University, United States). Cells were maintained in DMEM with pyruvate, GlutaMAX and 4.5 g of glucose/L (Gibco) supplemented with 10% FBS (Invitrogen) at 37°C and 5% CO_2_. The cells were passaged at around 80% confluence.

For immunoblotting cells were harvested at 80% confluence and proteins were extracted with RIPA buffer (Cell Signalling) containing complete protease and phosphatase inhibitor (Roche). Equal amounts of protein (15 μg) were fractionated by SDS-PAGE and transferred to nitrocellulose membranes. The membranes were blocked and incubated with primary antibodies overnight at 4°C, followed by incubation with HRP-conjugated secondary antibody (Invitrogen) for 1 h and development using an enhanced chemiluminescence kit (Millipore). The primary antibodies were: anti-Aurora (1:1000, Encor biotechnology), anti-Kif3a (Proteintech); anti-acetylated α tubulin (1:50000, Sigma); anti-integrin α5 (1:200, Santa Cruz), anti-cyclin D (1:1000, Millipore), anti-cyclin E (1:1000, Millipore), anti-integrin β1 (1:1000, Abcam) and HRP-conjugated anti-GAPDH (Proteintech). The following antibodies were from Cell Signalling: anti-phospho FAK (Tyr397), anti-phospho FAK (Tyr925), anti-total FAK, anti-phosphorylated ErK, anti-total ErK, anti-phosphorylated PKC, anti-Catenin, anti-phohsphorated GSK3β ser9, anti GSK3β, anti-HDAC6, anti-phosphorylated histone 3 and anti-phosphorylated PKC. Membranes were then incubated with HRP-conjugated secondary antibodies (Sigma, 1:3000) and developed with an enhanced chemiluminescence kit (Millipore).

### Immunohistochemistry and *in situ* Hybridization

Time-mated pregnant female mice received pulse BrdU (100 mg/kg) injections at E15.5 and coronal brain sections were prepared from electroporated pup brains at E16.5, P0 (20 μm thickness) and P7 (40 μm thickness) within a cryostat (Leica 1850). These sections were then processed for immunohistochemistry as described previously (Lang et al., 2016). The primary antibodies were: rabbit anti-GFP (1:800; Invitrogen), mouse anti-GFP (1:800, Invitrogen), rat anti-BrdU (1:500, Abcam), mouse anti-BrdU (1:200, BD), rat anti-Ki67 (1:100, BD), rabbit anti-Satb2 (1:500, Abcam), goat anti-CDP (1:200, Santa Cruz), rabbit anti-Ctip2 (1:300, Abcam), mouse anti-Tle4 (1:300, Santa Cruz) and goat anti-Sox5 (1:300, Santa Cruz), rabbit anti-Ki67 (1:1000, abcam). Species-specific Alexa Fluor 488 (1:500, Molecular Probe) or Alexa Fluor 594 (1:1000, Molecular Probe) conjugated secondary antibodies were used to detect primary antibodies. Heat mediated antigen retrieval (citrate buffer, pH = 6.0) was used to reveal BrdU incorporation. For birth-dating studies, double-labeling with anti-BrdU/anti-Ki67 was performed to examine the number of cells which exited from cell cycles.

For *in situ* hybridization, mouse brains (E12.5, E15.5, E17.5, P7 and 4 month) were fixed and sectioned at a thickness of 20 μm (for embryos) and 40 μm (for postnatal brains). Sections were mounted on poly-L-lysine coated slides and dried for 2 h at 55°C. Riboprobe complementary to 761 bp Ift172 cDNA was transcribed and subject to DIG RNA-labeling (SP6/T7; Roche Diagnostics, Ltd., United Kingdom). Hybridization was performed as described previously (Hu et al., 2014) and the signals were visualized using 5-Bromo-4-chloro-3-indolyl-phosphate (Boehringer Mannheim), and 4-nitroblue tetrazolium salt (BioRad) as substrates for alkaline phosphatase. The sequences of the primers were: Forward, 5′-AGTTGTCTCGGTCAGTGA-3′, Reverse, 3′-GCGGTGACGGTGTATAAG-5′.

### Primary Neuronal Culture and RT-qPCR

Primary neuronal culture was performed as described previously. Briefly, the cortices of E15.5 wildtype mouse embryos were dissected, trypsinized, and dissociated mechanically into single cell suspension. The cells were then plated at a density of 3 × 10^5^/ml into 6-well plates (Nunc) which were coated with poly-L-lysine (Sigma). The culture was maintained in Neurobasal medium (Invitrogen) supplemented with 2% B27 (Invitrogen) plus 2 mM glutamine (Sigma) at 37°C with 5% CO_2_. Twelve hours after plating, tissue debris was removed and the medium was renewed. On day 4 after plating, cells were transfected with shRNA813 or control shRNA combined with GFP (4 μg for each) using lipofectamine 2000 (Invitrogen). Transfection efficiency was determined with the percentage of GFP-positive cells/field and comparison was made between the control and knockdown groups.

Total RNA was isolated from cultured cells on day 6 using TRIzol reagent (Life Technologies) according to the manufacturer’s instruction. The cDNA was then synthesized from total RNA using the Taqman mRNA reverse transcription kit (Takara). RT-qPCR was performed with the Applied Biosystems 7500 Sequence Detection system, using iQ^TM^ SYBR Green Supermix (BioRad Laboratories, United States). The data were normalized to the geometric mean of housekeeping gene GAPDH values and calculated as 2^–ΔΔCT^ method. Sequences of the primers for RT-qPCR are summarized in Supplementary Table 2.

## Results

### WIM Cells Presented No Primary Cilium

**FIGURE 1:**
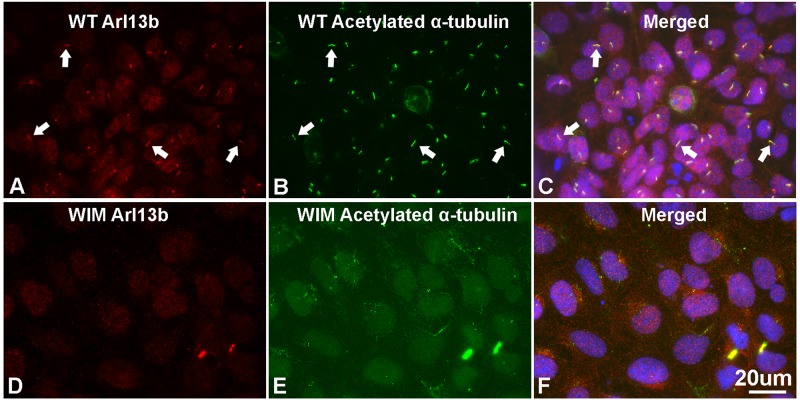
WIM cells have no primary cilium. **(A–C)** Most of WT MEF cells grew primary cilia after serum starvation for 21 h as evidenced by immuno-double staining with anti-Arl13b (red) and anti-acetylated α tubulin (green); Hoechst staining (blue) shows nuclei. Arrows show typical primary cilia. **(D–F)** As opposed to WT MEFs, serum starvation did not induce any growth of primary cilia in WIM cells. Bar = 20 μm.

**FIGURE 2:**
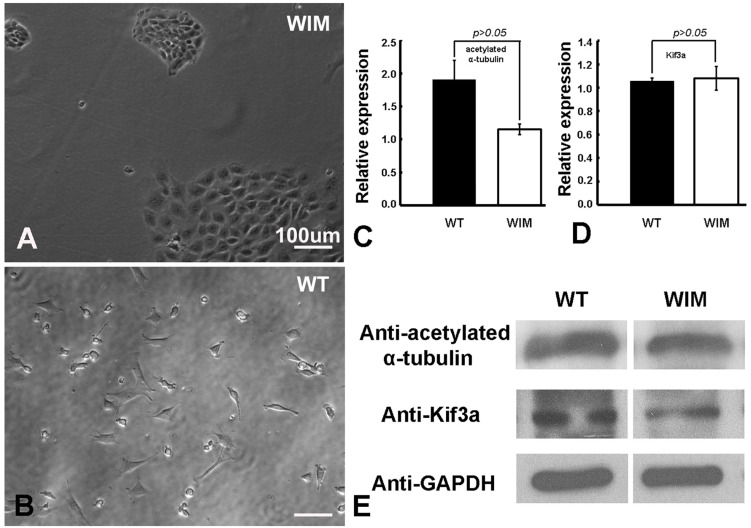
WIM cells **(A)** displayed a unique spreading growth pattern compared to WT cells **(B). (C–E)** WIM cells exhibited normal intracellular expression of acetylated α-tubulin (a core ciliary building block) and Kif3a (a key component of IFT complex B), indicating that WIM cells possess at least some other components for ciliary development and IFT complex function. Data analyzed using *t*-tests, *n* = 3.

To determine the roles of IFT172 in the growth and elongation of primary cilia, we immunostained WIM cells with anti-ARL13B and anti-acetylated α-tubulin. ARL13B is a small GTPase which localizes and facilitates the initiation of the primary cilium (Ruppersburg and Hartzell, 2014; Pruski et al., 2016), and acetylated α-tubulin is a key component of the ciliary axoneme. Compared to WT cells, WIM cells did not present any ciliary staining, displaying a complete absence of primary cilia (Figure 1). This highlights the essential roles of IFT172 in ciliary growth and maintenance. Further, we performed western blot examination to assess the cytoplasmic concentration of acetylated α-tubulin because it is also a core component of the cytoskeleton. Intriguingly, the *Ift172* mutation did not alter total acetylated α-tubulin expression (Figures 2C,E). In addition, there was no significant decrease in Kif3a levels (Figures 2D,E), another major component of IFT complex B (D’Angelo and Franco, 2009). Therefore, the lack of cilia on WIM cells is likely the direct consequence of the *Ift172* mutation. Interestingly, WIM cells exhibited a unique spreading growth pattern as they stayed in clumps (Figures 2A,B), suggesting stronger cell-to-cell adhesion or a lack of motility.

### Cilia Ablation Correlates With a Decrease in Electrotaxis

**FIGURE 3:**
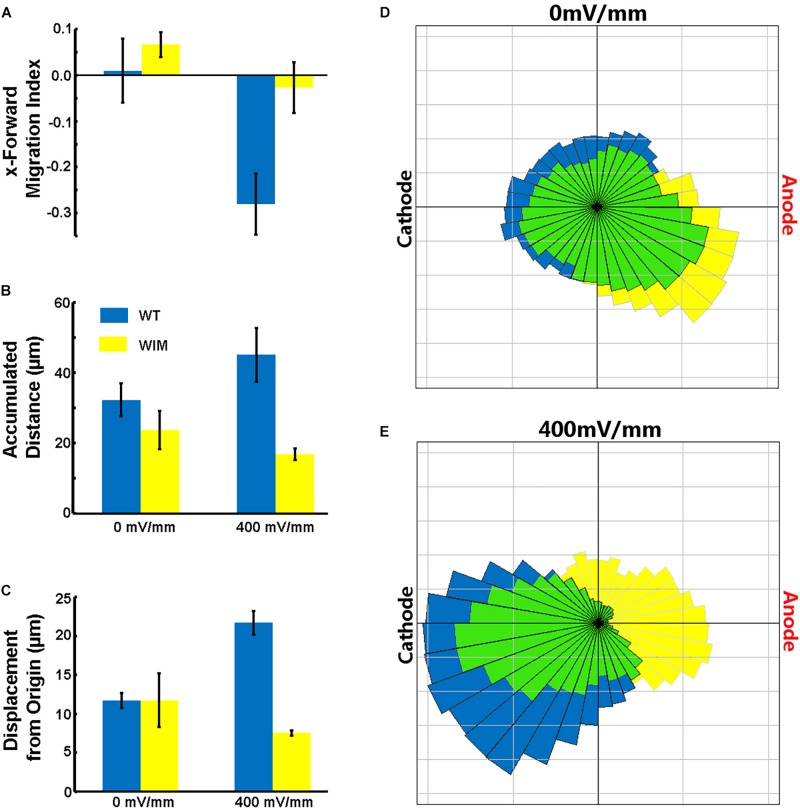
Effects of electric fields on MEF cells. **(A)** WT cells migrated toward the Cathode in the presence of the DCEF (*p* = 0.0293), while WIM cells did not show a significant response to the field. WIM cell accumulated distance (indicative of total cell motility) did not increase in the presence of the DCEF **(B)** nor did their displacement from origin (Euclidean distance) **(C)**, and with respect to both parameters there was a significant difference between the two genotypes (*p* < 0.01 in both instances). **(D,E)** Rose diagrams showing the proportion of cells with a net displacement in a particular direction; blue – WT, yellow – WIM, green – intersection of both WT and WIM cells. Data analyzed using a Two-Way ANOVA, with Tukey HSD *post hoc* analysis; *n* = 3 (minimum 100 cells per repeat).

The complete abrogation of primary cilia in WIM cells and our previous ARL13B findings (Pruski et al., 2016) prompted us to investigate whether the cells respond appropriately to a robust *in vitro* extracellular guidance cue. We choose direct current electric fields because the cellular response to electric fields does not depend exclusively on one specific receptor. Additionally, unlike chemotaxis gradients, an electric field is spatially uniform and its intensity is constant throughout the chamber – cells experience a constant cue, regardless of cell movement. Compared to WT cells, WIM cells had a much weaker response to the electrical guidance cue (Figure 3). As opposed to WT cells, WIM cells lacked any significant directional response to the electrical cue. In addition, the electrical cue did not cause the WIM cells to increase their general motility nor their guided directional movement. These abnormalities were not associated with any changes in gross tubulin dynamics as measured by the ratio of detyrosinated/tyrosinated α-tubulin (data not shown). Additionally, when WT cells were exposed to the electrical cue a significant increase in ciliation was observed (Supplementary Figure 1). Therefore, Ift172 might play important roles in electrically-directed migration.

### WIM Cells Featured Aberrant Cell Adhesion

**FIGURE 4:**
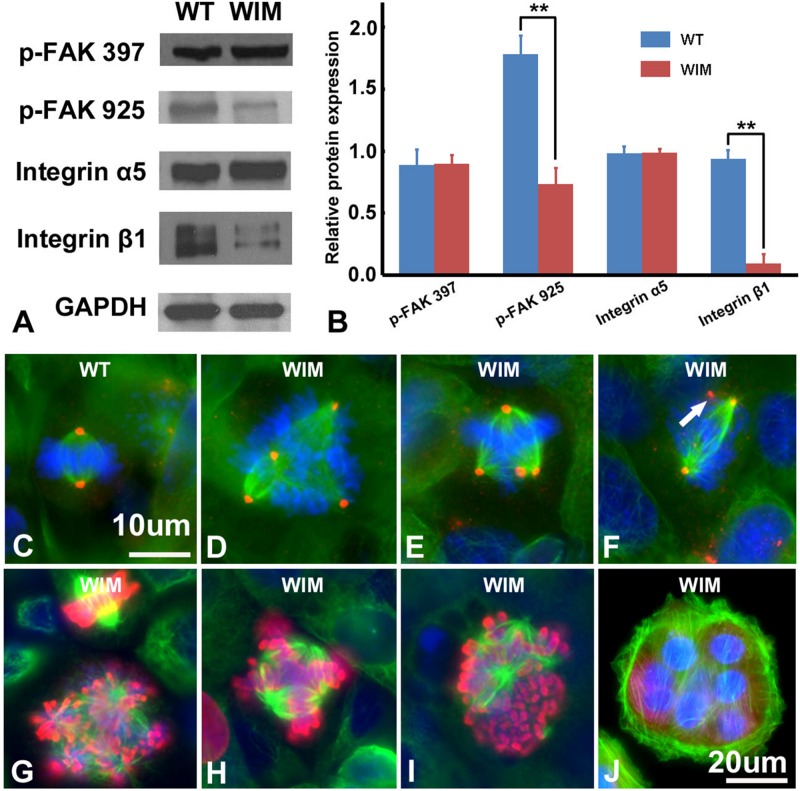
WIM cells featured aberrant cell adhesion and proliferation profiles. **(A,B)** Compared to WT cells, WIM showed significantly reduced expression of phosphorylated FAK925 and integrin β1. ^∗∗^*p* < 0.01; data analyzed using *t*-tests, *n* = 3. Unlike WT cells **(C)**, proportions of dividing WIM cells **(D–F)** exhibited eccentric microtubule organizing centers with multiple centrosomes (red). These centrosomes were either shared **(D,E)** which separated the cell’s chromosomes into multiple poles or functionally redundant (white arrowed in **F**). Red, anti-pericentrin staining; Green, anti-α tubulin staining. **(G–I)** The abnormal mitotic spindle was also demonstrated by double staining with anti-phosphorylated histone 3 (Red) and anti-α tubulin (in green). **(J)** A WIM cell-derived syncytium marked by anti-α tubulin staining. Bars are 10 μm in **(C–I)**, and 20 μm in **(J)**.

Compared to WT cells, cultured WIM cells grew in a unique spreading pattern, formed cell aggregations (Figures 2A,B), and required vigorous agitation for harvest during trypsin-facilitated cell passaging. Therefore, it was possible that they exhibited excessive adherence, which impeded migration. Together with a lack of change in microtubule dynamics (data not shown), this prompted us to examine the expression of several key adhesion molecules. Focal adhesion kinase (FAK) is a major signaling component of focal adhesion (FA) and co-ordinates cell migration via the balanced turnover between phosphorylated and dephosphorylated FA (Deramaudt et al., 2011). Our western blot results showed that WIM cells displayed normal expression of FAK phosphorylated at 397 but reduced levels of FAK phosphorylated at 925. Likewise, WIM cells presented normal levels of integrin α5 (Figures 4A,B) but significantly decreased levels of integrin β1 (Figures 4A,B), highlighting impairment of spreading-dependent cell migration (Davey et al., 1999).

### WIM Cells Had Altered Cell Cycle Profiles

**FIGURE 5:**
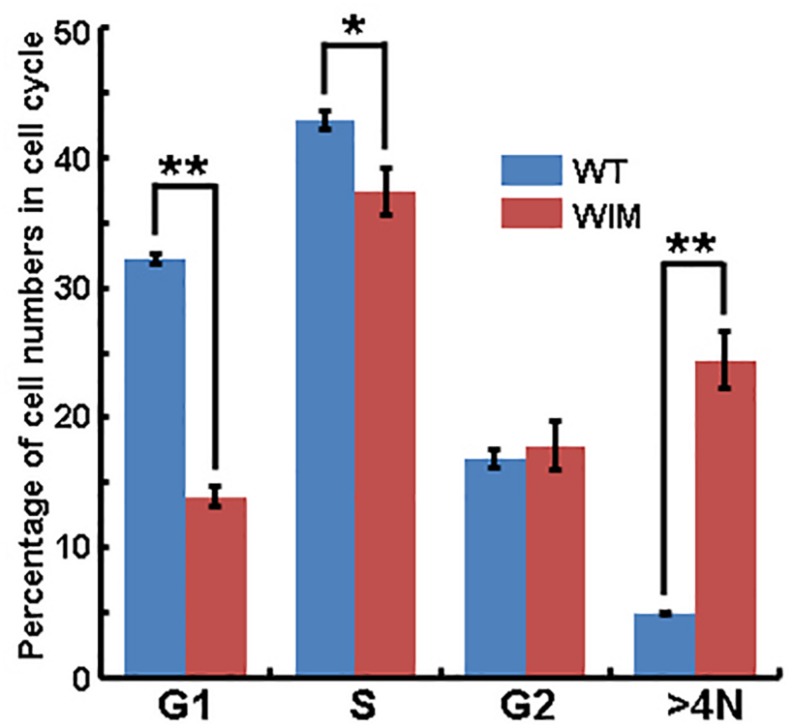
Flow cytometry analyze showed that significantly lower proportions of WIM cells were present in G1/G0 and S phase, but a higher proportion of cells contained four chromosome (>4N, as contrasted with WT cells). ^∗∗^*p* < 0.01; ^∗^*p* < 0.05; data analyzed using *t*-tests, *n* = 4 for WT and *n* = 3 for WIM.

**FIGURE 6:**
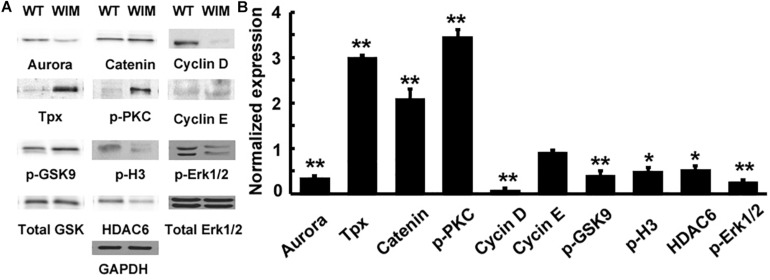
**(A)** IFT172 ablation disrupted multiple signaling pathways. **(B)** The protein expression was normalized and statistically compared between WT and WIM cells. WIM cells exhibited increased levels of TPX, p-PKC and β-catenin, but the other downstream targets including aurora, cyclin D, p-GSK9, p-H3, HDAC6, and p-Erk1/2 were all down-regulated. ^∗∗^*p* < 0.01; ^∗^*p* < 0.05; data analyzed using *t*-tests, *n* = 3.

As ciliary function was previously associated with effect of the cell cycle (Pruski et al., 2016), WIM cell proliferation was compared to the WT phenotype. WIM cells showed both a lower population size 4 days post-plating (Supplementary Figure 2) and a radically different cell cycle profile when compared to WT cells (Figure 5). The WIM group had fewer cells in the G1/G0 phase and S phase, with an increased number in the G2/M phase, and a remarkable proportion (∼25%) with more than 4 chromatids. Future karyotyping experiments might reveal whether particular chromosomes are more affected by this phenomenon. Immunocytochemistry revealed that dividing WIM cells often displayed multiple centrosomes (Figures 4D–F), leading to disorganized mitotic spindles and microtubule-organizing centers, which is likely to have contributed to the formation of frequently observed syncytia (Figure 4J). This observation was also confirmed by anti-phosphorylated histone 3 staining (Figures 4I,J), and a series of western blots of cell cycle related factors (Figure 6). WIM cells showed decreased expression of Cyclin D, HDAC6 and pH3/Ser10, while no changes were observed for Cyclin E. The cyclins play regulatory roles in cell cycle management, with lower levels of Cyclin D suggesting a problem in G1 to S phase transition (Adolphe et al., 2006). Both HDAC6 and pH3/Ser10 indicate changes in the histone status of WIM cells (Haggarty et al., 2003; Sawicka and Seiser, 2012; Awad et al., 2013). Additionally, a decrease in the levels of AURK A + B levels, as well as its increased association with TPX, were observed (Figure 6), with both of them associated with the centrosome, and AURKA being a known negative regulator of the primary cilium (Kufer, 2002; Yamada et al., 2010; Izawa et al., 2015). The primary cilium and the centrosome are closely linked, and the disruption of one can have profound effects on the other, amplifying perturbations in cell division and cell cycle management.

### WIM Cells Exhibited Disruptions in Signaling Pathways

Having established defects in both cell motility and cell cycle progression, we tested whether there were alterations in any molecular pathways (Figure 6). WIM cells showed changes in canonical WNT pathway components, with decreased levels of GSK3β phosphorylation at Ser9 and increased β-Catenin. pPKC levels were significantly increased, whilst ERK levels were decreased when compared to WT cells. Changes in signaling pathways are not surprising considering the role that primary cilia and the centrosome play in the reception and transduction of intercellular signals.

### *Ift172* Was Dynamically Expressed in Developing Mouse Cortices

**FIGURE 7:**
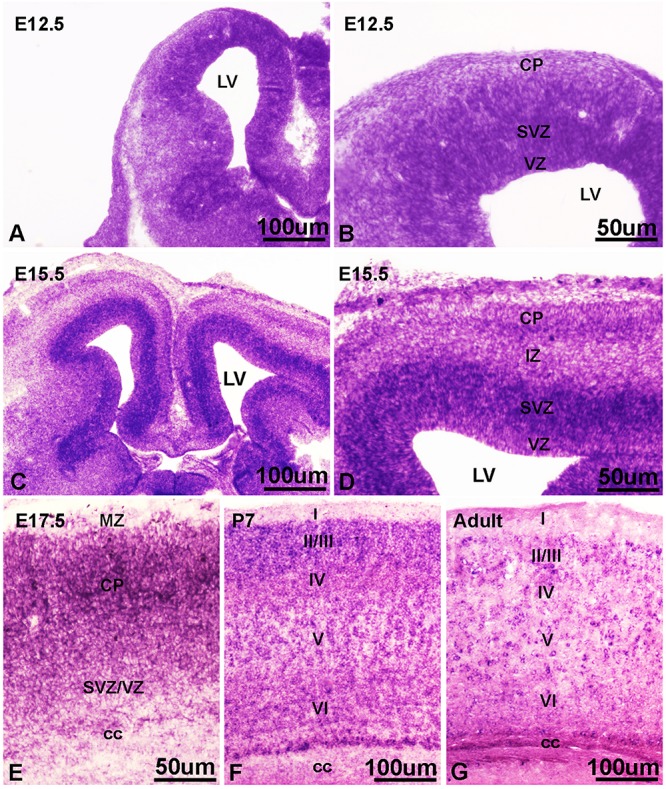
*In situ* hybridization showed dynamic expression of Ift172 in embryonic and postnatal mouse brains. **(A)** Ift172 transcripts were broadly expressed in E12.5 cortices. **(B)** Higher magnification view revealed strong localization of Ift172 in germinal zones (VZ and SVZ) in E12.5 cortex. **(C)** Ift172 mRNA was widely expressed in E15.5 cortex, with a preference toward germinal zones. **(D)** Higher magnification displayed intense hybridization signals in the VZ and SVZ regions. **(E)** Expression of Ift172 is more intense in the upper layer (cortical plate) than the bottom layer (VZ + SVZ) in E17.5 cortex. Expression of Ift172 broadly covers all cortical layers except layer I of P7 **(F)** and adult **(G)** mouse cortices. I–VI, sublayers of cortex. cc, corpus collasum. CP, cortical plate; IZ, intermediate zone; LV, lateral ventricle; MZ, marginal zone; SVZ, subventricular zone; VZ, ventricular zone; Bars = 100 μm in **(A,C,F,G)** and 50 μm in **(B,D,E)**.

Our *in vitro* data and findings implicating IFT172 in neurodevelopmental disorders (Halbritter et al., 2013; Schaefer et al., 2016), prompted us to investigate the *in vivo* roles of Ift172 during corticogenesis. We synthesized specific riboprobes against mouse *Ift172* mRNA and performed *in situ* hybridization in both embryonic and postnatal mouse brains. Mouse *Ift172* transcripts were detected broadly in E12.5 cortices, when active cortical neurogenesis takes place and brain lamination arises (Figures 7A,B). In E15.5 cortices, compared with intermediate zone and cortical plate, abundant hybridization signals were observed in the ventricular zone (VZ) and subventricular zone (SVZ) (Figures 7C,D). This distribution pattern highlights the potential regulatory roles of IFT172 in both neural progenitors and post-mitotic neurons that have exited the cell cycle and undergone migration and differentiation. In E17.5 cortices, hybridization signals were detected broadly (Figure 7E), and this distribution pattern extended into postnatal day 7 and adult brains (Figures 7F,G). Hybridization with sense probes also was carried out and no specific signals were detected (data not shown). These results show extensive and dynamic *Ift172* expression in both developing and postnatal cortices which strongly supports a regulatory role of Ift172 in neural network establishment and maturation.

### Knockdown of *Ift172* at E15.5 Significantly Perturbed Corticogenesis

In view of the intense expression of Ift172 mRNA in the germinal zone of developing cortices, especially at E12.5 and E15.5 (Figures 7A–D), we speculated that Ift172 may regulate the proliferation of neural progenitors, and thus, control the expansion of stem cell pools. To test this hypothesis, we analyzed the influence of *Ift172* knockdown, using *in utero* electroporation mediated gene-transfer, on the proliferation of neural progenitors.

The specificity of the shRNA813 and shRNA814 has already been validated by Sigma. To further confirm this, we took shRNA 813 as the example in primarily cultured mouse cortical neurons by RT-qPCR. The mRNA expression level of Ift88 and Ift20, another two members of Ift complex B family, was also measured as internal controls. As expected, transfection of shRNA813 significantly reduced the mRNA expression level of Ift172 in primarily cultured neurons, but had no effects to the expression of transcripts of Ift88 and Ift20 (Supplementary Figure 3).

Mouse *Ift172* shRNAs 813 and 814 were then injected individually into lateral ventricles of E15.5 mouse brains together with GFP, followed by *in utero* electroporation. The embryonic brains were collected at E16.5, 1 h after a BrdU pulse injection. To determine the proportion of transfected cells in S phase, immunostaining with anti-BrdU was performed and the ratio of GFP/BrdU double-labeled cells to the total number of GFP-containing cells was assessed. Our results revealed that delivery of *Ift172* shRNA813 (*n* = 5, *p* < 0.01) caused a significantly decreased number of GFP/BrdU double-labeled cells in cortical germinal zones compared with the control group (*n* = 5). Similarly, electroporation of *Ift172* shRNA814 (*n* = 3, *p* < 0.01) produced similar results (Supplementary Figure 4). As a high proportion of transfected cells were stuck in the corpus callosum, we performed confocal microscopic examination and found that they did not have any primary cilia (Supplementary Figure 5), showing successful *Ift172* silencing and resultant cilium abrogation. These results strongly indicate that silencing of *Ift172* in the developing brain significantly inhibits neural progenitor proliferation.

**FIGURE 8:**
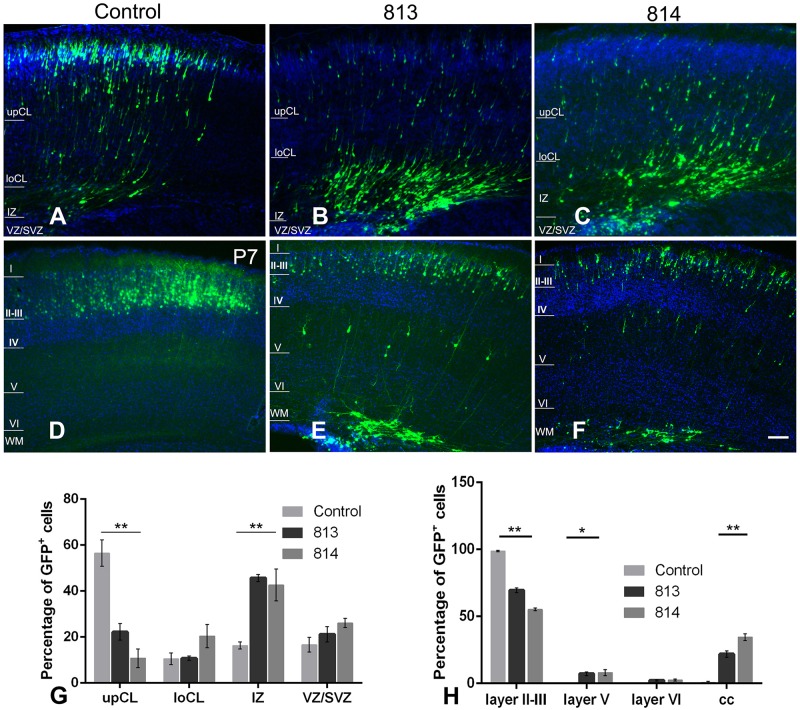
*In utero* silencing of ift172 significantly inhibited radial migration during corticogenesis. **(A)** In the control group, a high proportion of GFP-traced cells have migrated into superficial cortical layers and many others were en route at P0. **(B,C)** At P0, in both shRNA813 and 814 groups, most of GFP-traced cells were still positioned in intermediate cortical layers, with a few cells in the upper and lower cortical layers. **(D)** At P7, in the control group, almost all the GFP-labelled cells have completed the migration and are located in layers II–III. **(E,F)** At P7, although some cells finished the migration and were distributed in layers II–III in both shRNA813 and 814 groups, a good number of cells were still en route to the superficial layers or located in corpus callosum. **(G)** Percentages of GFP-traced cells in cortical layers of P0 mice. Both shRNA813 and 814 caused significantly reduced cell numbers in upper cortical layers, but increased cell number in intermediate cortical layers. ^∗∗^*p* < 0.01. 813, *n* = 4; 814, *n* = 4; control, *n* = 4. Two-way ANOVA plus Tukey *post-hoc* analysis was used. **(H)** Percentage of GFP-traced cells in cortical layers of P7 mice. Both shRNA813 and 814 caused a significant reduction of cell numbers in layers II–III, but increased cell numbers in layer V. ^∗∗^*p* < 0.01; ^∗^*p* < 0.05. 813, *n* = 6; 814, *n* = 3; control, *n* = 4. Two-way ANOVA plus Tukey *post-hoc* analysis was used. Bars = 200 μm in **C–H**.

We then investigated the influence of *Ift172* knockdown on radial migration, a precisely-coordinated process for fate-specification of cortical neurons. The distribution of GFP-traced cells was examined in P0 and P7 pup brains that received electroporation at E15.5. At P0, more than half of the transfected neurons in control brains had migrated into the upper cortical layers. The rest of the GFP-traced neurons were located in the lower cortical layers and presented typical radial glia morphology with a well-developed leading process toward, or reaching to, the cortical superficial layers (Figures 8A,G). In contrast, in both shRNA813 and 814 groups, the majority of GFP-traced neurons were positioned in lower cortical layers (Figures 8B,C,G). By P7, almost all the labeled neurons had completed migration and were located exclusively in layers II-III of control brains (Figures 8D,H). Strikingly, in both shRNA813 and 814 knockdown brains, only 69% and 55% GFP-positive neurons had reached layers II-III, respectively. In addition, a few GFP-positive neurons in layer V maintained the morphology of radially migrating cells whilst many others appeared “trapped” in layer VI or even in the corpus callosum with well-developed neurite arborization (Figures 8E,F,H). We then counted the number of GFP-positive cells in each layer and performed statistical analysis. Compared with control brains (*n* = 4), knockdown brains contained significantly less GFP-positive cells in superficial layers at P0 (for 813, *n* = 4, *p* < 0.001; for 814, *n* = 4, *p* < 0.001) and P7 (for 813, *n* = 6, *p* < 0.001; for 814, *n* = 3, *p* < 0.001), but higher proportions in deeper layers at P0 (for 813, *n* = 4, *p* < 0.001; for 814, *n* = 4, *p* < 0.001) and P7 (for 813, *n* = 6, *p* < 0.001; for 814, *n* = 3, *p* < 0.001), indicating dramatically disrupted radial migration.

To further examine the fate specification of neurons with Ift172 silencing in mouse cortices, we performed immunofluorescent double labeling study in P7 brain slices using layer-specific markers. In control group, all the GFP-tagged neurons have migrated into layer II/III and co-expressed Satb2, a highly conserved nuclear protein specific for callosal pyramidal neurons. Interestingly, in knockdown groups, the majority of GFP-tracked neurons, regardless of their location, still co-expressed Satb2 (Supplementary Figures 6, 7). This result indicates that though Ift172 has little effects on cell fate determination, radial migration was overtly disturbed.

## Discussion

Being a component of the IFT complex B and of the BBSome, human IFT172 deficiency can cause several discrete conditions, such as short rib thoracic dysplasia, retinitis pigmentosa, and BBS. BBS is a collection of autosomal inherited conditions which often present neurological symptoms in children, including impaired gait, movement coordination and learning deficits. The underpinning mechanisms remain largely unclear. Although *Ift172* mutation causes loss of the primary cilium (Figure 1) and severe brain patterning deficiency in mice, no data were available previously regarding its precise roles in cortical development as both *Ift172 Wimple* mice and knockout mice die at an early (E11.5) embryonic stage (Huangfu et al., 2003; Huangfu and Anderson, 2005; Ocbina and Anderson, 2008; Gorivodsky et al., 2009). Using cells derived from *Wimple* mice and *in utero* gene manipulation, we observed a range of cellular behavioral abnormalities both *in vitro* and *in vivo*. To our knowledge, this is the first report that primary cilium removal caused by *Ift172* mutation regulates corticogenesis by governing cell proliferation and migration. We conclude that IFT172 is essential for ciliary health and plays a profound role in the morphogenesis of the cerebral cortex. Yet we also wish to highlight that the aberrations are the likely result from functional ciliary defects, consistent with ciliary studies of other genes (Oh and Katsanis, 2013; Fogelgren et al., 2014; Pruski et al., 2016), though, as suggested by, *Ift88* studies, cilia independent effects might be present (Boehlke et al., 2015).

### IFT172 Is Important for Cell Function

Although IFT172 was first identified a decade ago, with the exception of Hedgehog signaling, there has been no data elucidating its related pathways (Huangfu et al., 2003). In our study, down-regulation of Ift172 altered the expression of many signaling pathways, though it is likely that the observed changes are due to a general lack of the cilium with further knock-on effects on the centrosome, as opposed to a direct influence of IFT172 disruption. Yet, considering the wide range of ciliopathies, the genes that contribute to them and their phenotypic manifestations (Gerdes et al., 2009; Tobin and Beales, 2009; Norris and Grimes, 2012; Valente et al., 2014), some gene specific effects are likely to be present.

Cilia are known to facilitate the transition from canonical WNT signaling to non-canonical WNT signaling (Basten and Giles, 2013). Consistent with having dysfunctional cilia, WIM cells had alterations in WNT signaling components. β-Catenin levels are known to both increase and decrease in response to ciliary mutations (Oh and Katsanis, 2013) and our study revealed an increase in β-Catenin levels (Figure 6) indicating an increase in canonical WNT pathway activity. Paradoxically, GSK3β phosphorylation on Ser9 decreased (Figure 6), indicative of a reduction in canonical WNT signaling (Fukumoto et al., 2001; Chen et al., 2014; Llorens-Martín et al., 2014). This suggests that some aspects of these changes originate from sources outside the WNT pathway, e.g., due to cell clumping (Figure 2). Cell-cell adhesions could reduce cell proliferation, but this is usually associated with a decrease in WNT dependent β-Catenin activity (Stockinger et al., 2001; Harris and Peifer, 2005). Therefore, the reasons for these changes in the WNT pathway seem complex and, for the moment, idiopathic. Nevertheless, these changes are unlikely to have caused the lower WIM cell population levels 4 days post-plating.

Lower Cyclin D expression in WIM cells (Figure 6) might have led to a problem in the transition from the G1 phase to the S phase. Yet, the proportion of WIM cells in S phase to those in G1/G0 is higher than in WT cells (Figure 5). A possible explanation is that a proportion of cells within the > 4 chromatid group is biochemically in the G1/G0 group and are somehow inhibited from transitioning into the S phase due to chromatid or centrosome problems (Figures 4C–J), causing the cells to stop at one of the cell cycle checkpoints. Furthermore, reduced levels of integrin β1 (Figure 4B) have been implicated in the prevention of spreading-dependent cell proliferation (Davey et al., 1999), and thus may also contribute to the slow WIM cell proliferation. Additionally, the decrease in phosphorylation of H3 at Ser10 and in HDAC6 levels (Figure 5) further highlights the inability of WIM cells to manage chromatids. Activity changes of AURK A + B (Hannak et al., 2001; Bayliss et al., 2003; Plotnikova et al., 2012, 2015; Izawa et al., 2015) were also observed (Figure 5). Hence, in WIM cells, the ablated cilium correlates with dysfunctional centrosome physiology and has a detrimental effect on cell division. These centrosomal changes are likely to influence cell migration as well as the establishment of cell identity during brain development (Yamada et al., 2010; Bakircioglu et al., 2011; Paridaen et al., 2013).

ERK changes were associated previously with ciliary mutations, but both decreases (Higginbotham et al., 2012) and increases (Ma et al., 2013) in phosphorylation were reported. ERK signaling is crucial for processes ranging from the cellular stress response and cell polarization (including that of the Golgi apparatus and of the centrosome) during migration (Lu and Xu, 2006; Bisel et al., 2008). This is particularly important as cell polarity proteins are key for ciliary health, but conversely their action is also dependent on cilia mediated signaling (Pruski et al., 2016; Hua and Ferland, 2018), and so defects in cell polarity proteins might have self-enhancing effects. The decrease in ERK phosphorylation in WIM cells (Figure 5) might be a result of the WIM cells having a decreased ability to sense the growth factors associated with cilia, resulting in reduced cell proliferation (Schneider et al., 2005, 2010; Sotak and Gleeson, 2012), and which might translate to reduced cell migration capabilities, especially *in vivo*, where a range of chemical, physical and electrical cues might guide the moving cells. These results, together with an increase in ciliation during migration of WT MEF cells (Supplementary Figure 2) strengthen the hypothesis that cilia might not so much be responsible for sensing the direction of the migratory cue, as for assessing the appropriateness of the extracellular conditions for migration (Higginbotham et al., 2012; Pruski et al., 2016).

### IFT172, a New Link Between Ciliopathies and Neurodevelopmental Disorders?

**FIGURE 9:**
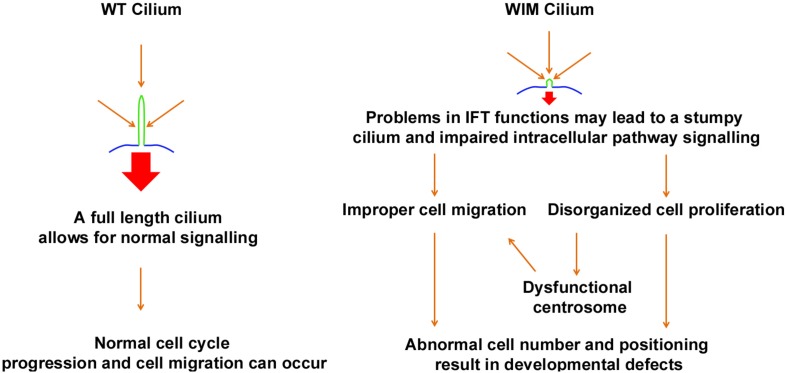
Schematic diagram outlines the proposed influence of primary cilium to fundamental cell biology and neurodevelopmental disorders.

Many children with BBS manifest learning problems and cognitive deficiency with an unclear underlying mechanism. Hence BBS, a ciliopathic condition with central nervous system phenotypes, has long been proposed to share some common mechanisms with mental disorders (Kamiya et al., 2008; M’hamdi et al., 2014). Ift172 mutation disrupts ciliary integrity, causing alterations in multiple intracellular signaling pathways. This undermines the capacity for cell proliferation and migration both *in vitro* and *in vivo*, as such being detrimental to proper neural network establishment and maturation (Figure 9).

Notably, the spatiotemporal expression pattern of Ift172 is similar to that of Ulk4, a rare risk factor we uncovered recently which is implicated in neocortical development and mental illness (Lang et al., 2014, 2016). Although Ulk4 has not yet been implicated in primary cilia function, it was shown to affect both the structure and function of motile cilia. Moreover, motile cilia have been postulated to affect the development of hydrocephalus via effects on primary cilia, hence such a synergistic effect should not be ruled out in other neurodevelopmental disorders (Lang, 2006; Carter et al., 2012; Sotak and Gleeson, 2012). *Disrupted-in-schizophrenia 1 (DISC1)* is one of the best-studied candidate genes for human mental disorders. Interestingly, *DISC1* also locates to and regulates primary cilia function (Marley and von Zastrow, 2010). *DISC1* also localizes at the centrosome and, together with BBS4, regulates it (Morris, 2003; Bradshaw et al., 2008; Kamiya et al., 2008). In addition, many other psychiatric risk genes also converge on this tiny organelle (Marley and von Zastrow, 2012) and a wide range of phenotypes is associated both with ciliopathies and the aberrant development of nervous system (Valente et al., 2014). Interestingly, a shortening of primary cilia by conditional Arl13b knockout re-shaped interneuronal connectivity and may underpin human mental disorders (Guo et al., 2017). In addition, a recent study illustrated clearly that primary cilia formation was diminished in patients with schizophrenia and bipolar disorders (Muñoz-Estrada et al., 2017). Our data strengthen the case for both ciliary involvement in and the developmental nature of mental illness (Pruski and Lang, 2019).

International human tissue banks lack brain samples from short rib thoracic dysplasia and BBS patients, therefore we could not perform pathological examination of human brain tissue with IFT172 mutation. However, previous studies of BBS patients have identified broad developmental brains defects. Quantitative fMRI of BBS patients as young as 14 years old showed globally thinned gray matter and a smaller hippocampus, which may underpin the learning difficulties and behavioral problems observed in BBS (Baker et al., 2011). Intriguingly, several established BBS mouse models also have thinned cerebral cortices, reduced corpus striatum/hippocampi and retinal degeneration (Davis et al., 2007). In addition, knockdown of subtypes of BBS proteins (Kamiya et al., 2008) or associated binding partners (Ishizuka et al., 2011) also cause migration defects of cortical neurons, which are consistent with our observation. Migration defects are not restricted to neurons because BBS patients and mice have similar craniofacial dysmorphologies and Hirschsprung’s disease due to dysfunctional neural crest migration (Tobin et al., 2008). IFT172 mutation can cause sporadic retinal degeneration and BBS in humans. Intriguingly, recapitulation of *Ift172* mutation (Bujakowska et al., 2015) or conditional *Ift172* knockout (Gupta et al., 2018) could lead to similar retinopathy in mice.

Based on these observations, we assume that it is highly likely that the cortical development deficits we have observed may also exist in human patients. Yet, further study is necessary to elucidate both the extent of ciliary role in particular neurodevelopmental disorders, as well as the impact of IFT172 function in human patients. To this end our current study is somewhat limited due to the severity of the ciliary phenotype caused by the null mutation, that would most likely prove fatal in early human development.

## Conclusion

Though IFT172 is strictly associated with the cilium, this study showed its mutations caused a wide range of cellular phenotypes, ranging from decreased motility and directed migration, to cell cycle changes and alterations in various cellular pathways, including canonical WNT and ERK signaling. The severity of these changes has been highlighted by the fact that IFT172 mutation can be embryonically lethal, and therefore any human pathology associated with IFT172 defects is likely to be caused by less severe genetic defects. The wide spectrum of intracellular changes associated with this ciliary mutation makes it unlikely that pharmacological treatment will be a feasible option for the treatment of diseases caused by dysfunctional IFT172 and by faulty cilia in general. Better understanding of ciliary function and the role of its specific components will benefit regenerative medicine and biotechnology where fine manipulation of cellular physiology is a key requirement. As highlighted by Ift172 knock-down in the developing brain, understanding cilia function in neurodevelopment could be key in designing better neuroregenerative interventions.

## Data Availability Statement

All datasets generated for this study are included in the article/Supplementary Material.

## Ethics Statement

All the animal care and experimental protocols were reviewed and conducted at Tongji University in compliance with local policies and regulations.

## Author Contributions

BL, Y-QD, CM, AR, and DS conceived the project. MP and LH performed the majority of the experiments. CY, YW, J-BZ, LZ, YH, and BL assisted with some of the experiments. MP, LH, CY, BL, Y-QD, CM, and AR analyzed the data. MP and LH performed the statistical analysis. MP, LH, BL, Y-QD, CM, and AR drafted the manuscript. All authors had an opportunity to comment on the final manuscript version.

## Conflict of Interest

The authors declare that the research was conducted in the absence of any commercial or financial relationships that could be construed as a potential conflict of interest.
