# Supplementary material for: Roles for IFT172 and Primary Cilia in Cell Migration, Cell Division, and Neocortex Development
Source: Front Cell Dev Biol. 2019 Nov 26;7:287. doi: 10.3389/fcell.2019.00287 (PMC6890611; doi:10.3389/fcell.2019.00287)
Supplement: Supplementary file 1 [file Data_Sheet_1.pdf]

Roles for ift172 and primary cilia in cell migration, cell division and neocortex development---Supplementary Materials

Michal Pruski[1,2,3,4,5*§], Ling Hu[3,5*], Cuiping Yang[4], Yubing Wang[4], Jin-Bao Zhang[6], Lei Zhang[4,5], Ying Huang[3,4,5], Ann M Rajnicek[5], David St Clair[5], Colin D McCaig[5], Bing Lang[1,2,5#], Yu-Qiang Ding[3,4#]

1 Department of Psychiatry, the Second Xiangya Hospital, Central South University, Changsha, Hunan 410011, China

2 National Clinical Research Center for Mental Disorders, Changsha, Hunan, China.

3 State Key Laboratory of Medical Neurobiology and MOE Frontiers Center for Brain Science, Institutes of Brain Science, Fudan University, Shanghai, 200032, China

4 Key Laboratory of Arrhythmias, Ministry of Education, East Hospital, Department of Anatomy and Neurobiology, Collaborative Innovation Centre for Brain Science, Tongji University School of Medicine, Shanghai 200092, China

5 School of Medicine, Medical Sciences & Nutrition, Institute of Medical Sciences, University of Aberdeen, Aberdeen AB25 2ZD, Scotland, United Kingdom

6 Department of Histology and Embryology, Institute of Neuroscience, Wenzhou Medical University, Wenzhou, Zhejiang 325035, China

* - These authors contributed equally

§: Current address: Critical Care Laboratory, Critical Care Directorate, Manchester Royal Infirmary, Manchester University NHS Foundation Trust, Manchester M13 9WL, United Kingdom

# - Address correspondence to Dr. Bing Lang at bing.lang@csu.edu.cn, or Dr. Yu-Qiang Ding at dingyuqiang@vip.163.com.

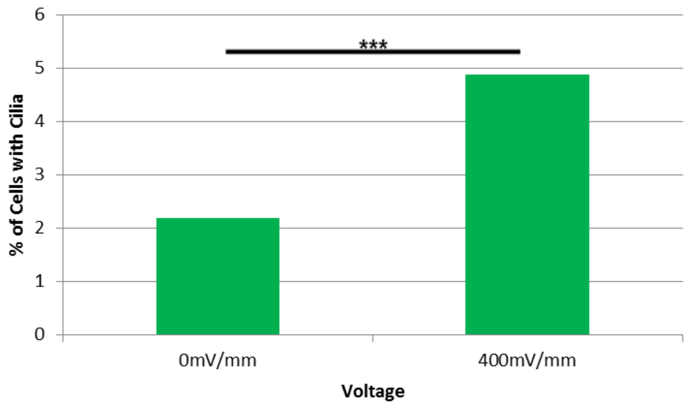

Supplemental Figure 1. The effect of a direct current electric field on the abundance of primary cilia. The electric field caused a rise of the percentage of cells showing primary cilia in the presence of serum in the medium. Chi-square test P<0.0001, data collated from 3 separate repeats of each condition, with an average of 857 cells assessed per repeat. A Chi-square test is a comparison of the whole populations of cells (ciliated and non-ciliated) in both voltage conditions (0 and 400mV/mm); the graph shows the proportion of ciliated cells in both conditions for ease of understanding the magnitude of the effect of an electric field on cell ciliation.

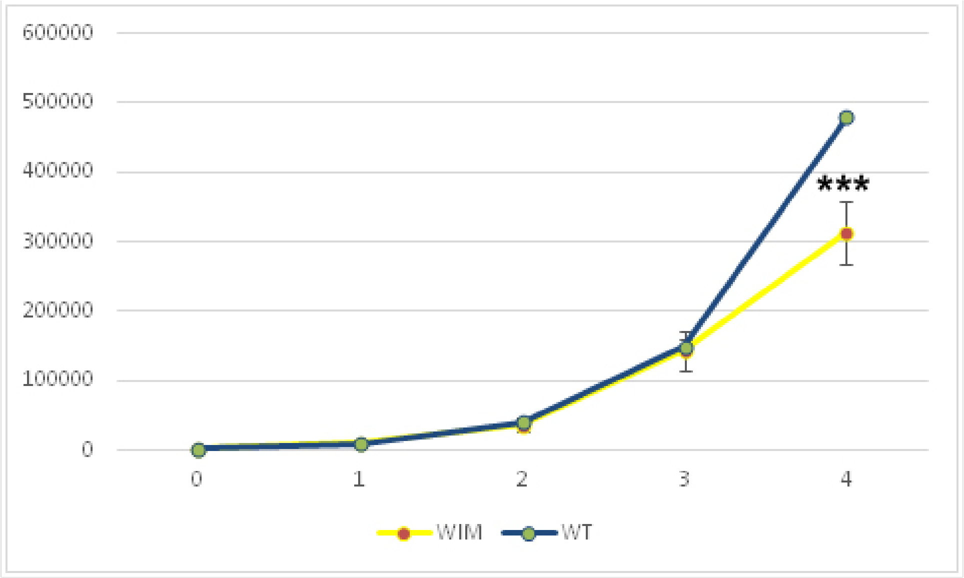

Supplemental Figure 2. Population growth of the two MEF lines. WIM cell cultures were observed to proliferate slower than WT cells. Data points indicate mean population levels; error bars show SEM (error bars on some points are smaller than the data points). The general effect of genotype is significant (p = 0.0054), as is the effect of days (p < 0.0001), and the interaction of days and genotypes (p = 0.0003). Differences between specific cell lines are indicates on the graph *p < 0.0001, n = 3 (Two-Way ANOVA, with Tukey HSD).

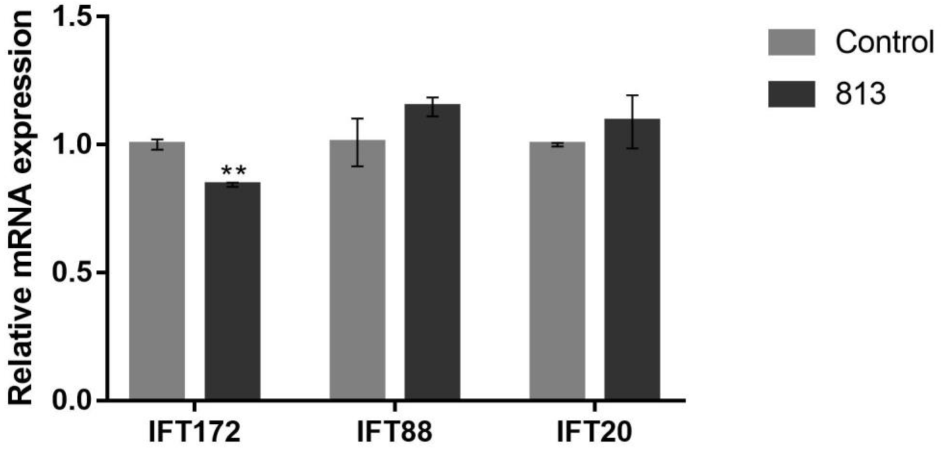

Supplemental Figure 3. RT-qPCR study shows that shRNA813 specifically reduces the mRNA expressing level of Ift172 in primarily cultured neurons, and has no effects on the expression of mRNA transcripts of Ift88 and Ift20. **, $p < 0.01$ (n=3).

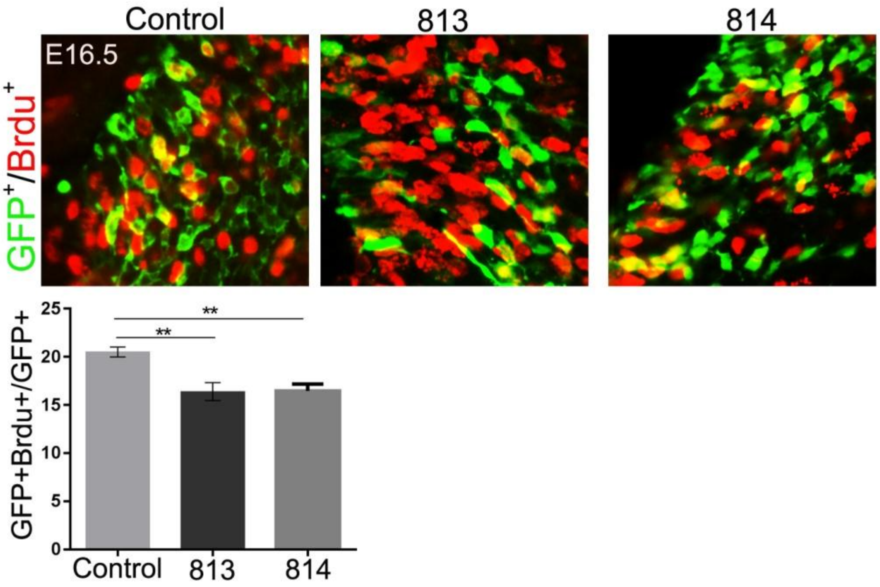

Supplemental Figure 4. Compared with controls (n = 5), both shRAN813 (n = 5, $p < 0.01$) and 814 (n = 3, $p < 0.01$) knockdown groups display decreased cell proliferation as revealed by a reduced BrdU incorporation ratio (GFP+BrdU+/GFP+). Data were analyzed using a Two-Way ANOVA, with Tukey HSD post hoc method.

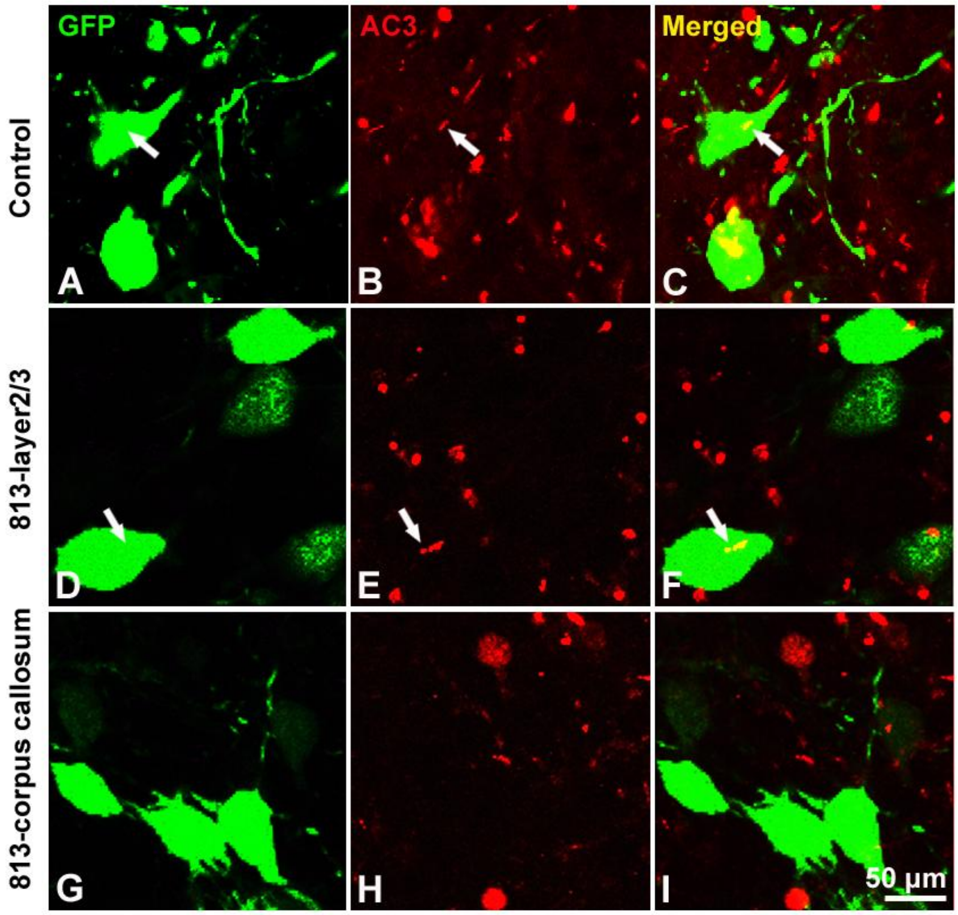

Supplemental Figure 5. Confocal image scanning reveals the existence of primary cilia (white arrowed) in neurons which have successfully migrated into layers II-III in both control (A-C) and shRNA813-knockdown brains (D-F). On the contrary, neurons which are "trapped" within the corpus callosum in shRNA813 knockdown group, do not have cilia (G-I). Bar = 50 μm.

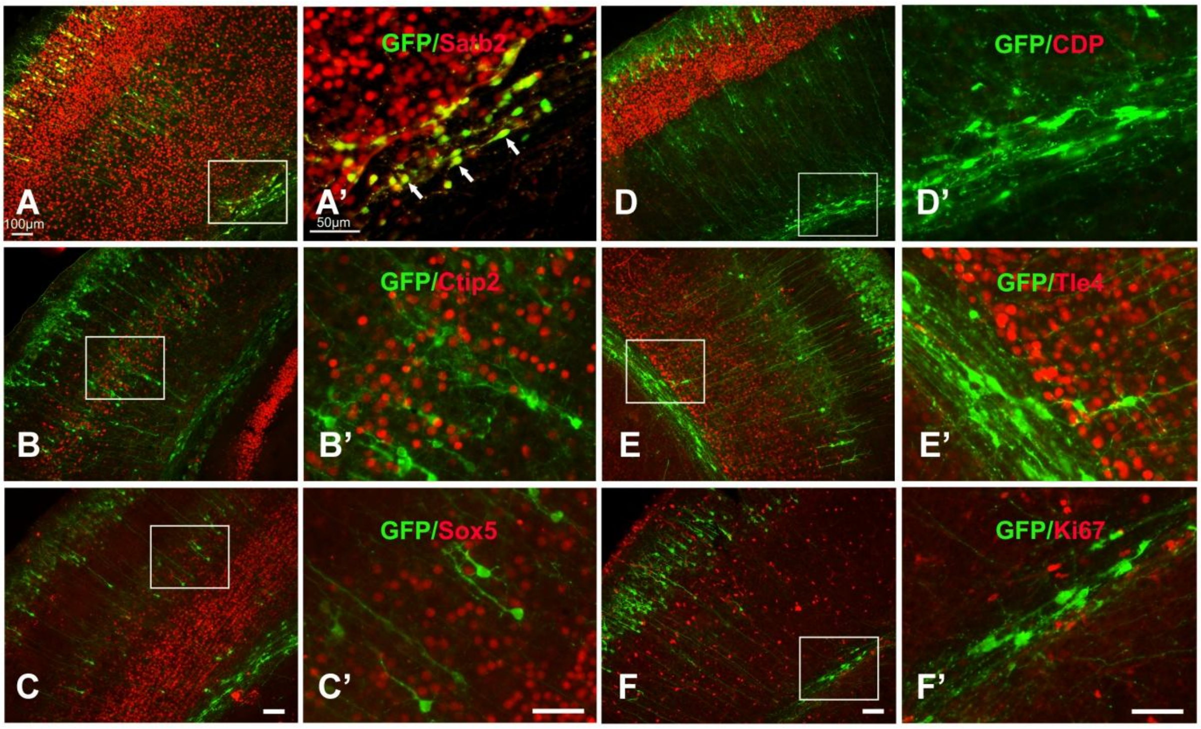

Supplemental Figure 6. (A-A′) Confocal image scanning reveals that the majority of GFP-tagged neurons in shRNA813 silencing group, regardless of location, co-express Satb2. (**B-F′**) GFP-positive cells do not express Ctip2 (**B-B′**), Sox5 (**C-C′**), CDP (**D-D′**), Tl4 (**E-E′**), and proliferating marker Ki-67 (**F-F′**). **(A′-F′**) are enlarged images of boxed areas in (**A-F**). White arrows in A′ show representative GFP/Satb2 double-labelling cells. Scale Bars in A-F = 100 μm, in A′-F′ = 50 μm.

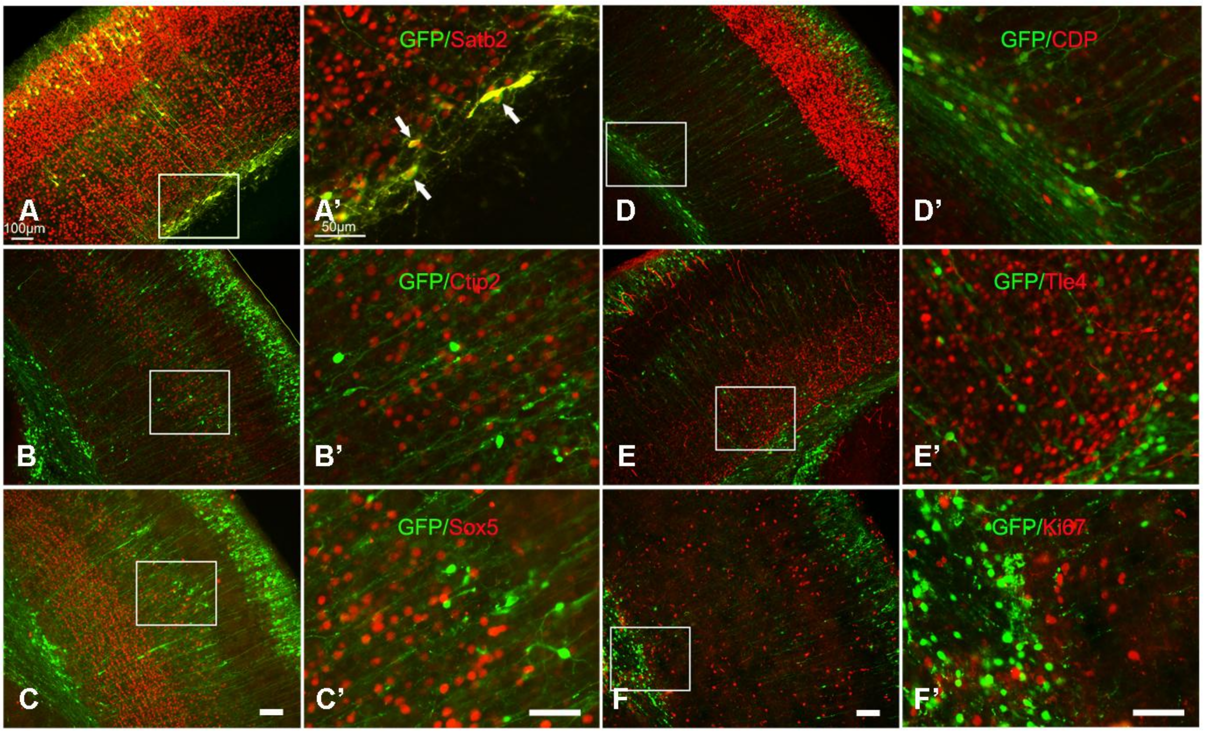

Supplemental Figure 7. Consistent with shRNA813 group, the majority of GFP-positive neurons in shRNA814 group co-express Satb2 (**A-A′**), instead of Ctip (**B-B′**), Sox5 (**C-C′**), CDP (**D-D′**), Tle4 (**E-E′**) and Ki-67 (**F-F′**). **(A′-F′**) are enlarged images of boxed areas in (**A-F**). White arrows in A′ show representative GFP/Satb2 double-labelling cells. Scale Bars in A-F = 100 μm, in A′-F = 50 μm.

Supplemental table 1: Detailed information of shRNA813 and shRNA814

Clone ID	TRCN0000079813	TRCN0000079814
VALIDATE_METHOD	SYBR	SYBR
VALIDATE_KNOCKDOWN	94%	91%
VALIDATE_CELLLINE	Hepa 1-6	Hepa 1-6
VALIDATED	Yes	Yes
geneDesc	intraflagellar transport 172 homolog (Chlamydomonas	intraflagellar transport 172 homolog (Chlamydomonas
targetTaxonId	10090	10090
NCBI_gene Id	67661	67661
nmId	NM_026298	NM_026298
TRC VERSION	1	1
Clone Name	NM_026298.4-2716s1c1	NM_026298.4-4704s1c1
oligoSeq	CCGGGCGGCCATCAACCACTATATTCTCGAGAATATAGTGGTTGATGGCCGCTTTT TG	CCGGGCTGCTGATCTCTCATTACTACTCGAGTAGTAATGAGAGATCAGCAGCTTTTTG

All the information was summarized from Sigma and the Broad Insitute websites

Supplemental Table 2 Pairs of Primer sequence used for RT-qPCR

Primer name	Primer sequence
ift88 forward:	TTGCGAGGCTCTGCATTTGA
ift88 Reverse:	ACAACTGTTGGCAATACAGCTTT
ift20 Forward:	AGGCAGGGCTGCATTTTGAT
ift20 Reverse:	CAAGCTCAATTAGACCACCAACA
IFT172 Forward:	AGATCAGGTGTCCAGGGAATATG
IFT172 Reverse:	CAGCGGTGCAACTCTTGGT
GAPDH-F Forward:	CTCATGACCACAGTCCATGC
GAPDH Reverse:	CACATTGGGGGTAGGAACAC
